# Supplementary material for: An increase in neural stem cells and olfactory bulb adult neurogenesis improves discrimination of highly similar odorants
Source: EMBO J. 2019 Jan 14;38(6):e98791. doi: 10.15252/embj.201798791 (PMC6418468; doi:10.15252/embj.201798791)
Supplement: Supplementary file 1 — Appendix [file EMBJ-38-e98791-s001.pdf]

APPENDIX

An Increase in Neural Stem Cells and Olfactory Bulb Adult Neurogenesis Improves Discrimination of Highly Similar Odorants

Sara Bragado Alonso, Janine Reinert, Nicolas Marichal, Simone Massalini, Benedikt Berninger, Thomas Kuner and Federico Calegari

SUPPLEMENTARY MATERIALS AND METHODS

Animals and treatments p. 2

In situ hybridization p. 2

Whole-mount immunostaining p. 3

Image acquisition and quantifications p. 3

Electron microscopy p. 4

Image processing and analysis p. 4

Electrophysiology p. 5

Olfactometry data acquisition and analysis p. 7

SUPPLEMENTARY MATERIALS AND METHODS

Animals and treatments The 4D– and 4D+ lines were generated crossing the individual heterozygous lines described (Nonaka-Kinoshita et al., 2013; Imayoshi et al., 2008; Belteki et al., 2005) to first obtain *nestin*$^{CreERT2+/+}$::*ROSA26*$^{rtTA-flox+/+}$::*Tet*$^{4D-RFP+/-}$ mice with only the latter allele being kept as heterozygous. This line was then used for genetic background homogenization by inbreeding for >5 generations after which triple homozygous 4D– and 4D+ were selected as founders of the two lines. From these, 8 week old mice from different litters were used for all experiments except for cell morphometry and electrophysiology that further required the crossing of the triple lines with *RCE*$^{GFP-flox+/+}$ mice (Miyoshi et al., 2010). In these settings, multiple heterozygous lines were used given that the two alleles of the *Rosa26* locus carried different transgenes (rtTA and GFP). No significant difference in phenotypes, BrdU incorporation, neurogenesis and increase in neurons was observed when comparing the quadruple heterozygous 4D with the previously described triple homozygous lines. Tamoxifen (Sigma) was dissolved in corn oil (1:10) and administered by gavage once a day for 3 days starting 1 week before administration of 9-tert-butyl doxycycline (Echelon Biosciences), which was dissolved in DMSO, dH_2O and corn oil (1:1:10) and injected subcutaneously twice a day for 4 days. BrdU (Sigma) and EdU (Sigma) were dissolved in PBS and administered intraperitoneally.

In situ hybridization After permeabilization with RIPA buffer (Sigma-Aldrich) and Tween 0.1% in PBS sections were incubated overnight at 70°C with digoxigenin-labeled RNA probes (400 ng/ml) in hybridization buffer (50% formamide, 0.1% tween-20, 10% dextran, 1 mg/ml yeast RNA, 100 mg/ml

heparin, 1x Denhardt's solution, 0.1% CHAPS and 5 mM EDTA). Washing was performed with 50% formamide, 0.1% tween-20 in 5x and 2x sodium citrate at 70°C and then at RT with 100 mM maleic acid, 150 mM NaCl, 0.1% Tween-20. Samples were blocked for 1h with maleic acid and 10% blocking reagent (Roche), incubated overnight at 4°C with anti-digoxigenin conjugated with alkaline phosphatase (Table 1), washed (100 mM Tris, 100 mM NaCl, 50 mM $MgCl_2$, 0.1% Tween-20, pH 9.5) and revealed with BM purple (Roche) at 37°C

Whole-mount immunostaining Whole OB were perfused with 4% PFA and dehydrated with methanol series (from 20% to 100%) 1h each and incubated overnight with 66% dichloromethane/33% methanol. Samples were bleached 12h with 5% H_2O_2 in methanol at 4°C, rehydrated with methanol series (from 80% to 20%) 1h each and washed twice with PTx.2 (0.2% TritonX-100 in PBS) for 1h. Permeabilization (80% PTx.2, 10% DMSO, 0.3 M glycine) and blocking (84% PTx.2, 6% donkey serum, 10% DMSO) were performed for 2 days each at 37°C. Incubations with primary and secondary antibodies (as above) were performed at 37° in PTwH (10% PBS, 0,2% Tween-20 and 0,1% heparin 10mg/ml) with 5% DMSO/3% donkey serum or 3% donkey serum for 8 and 10 days, respectively, with 4-5 washes with PTwH each. Clearing was performed by dehydration with methanol series (from 20% to 100%) 1h each and 3h incubation with 66% dichloromethane/33% methanol, twice 15 min with dichloromethane and dibenzyl ether.

Image acquisition and quantifications Immunohistochemistry and in situ hybridization were acquired with an automated Zeiss ApoTome or confocal

microscope (LSM 780) (Carl Zeiss). Clarity images were acquired by sample excitation with bidirectional triple light sheet technology using a ultramicroscope (LaVision BioTec, Germany) and fluorescence detected with a 16 bit sCMOS Andor Neo camera (Andor Technology Ltd, Northern Ireland) perpendicular to the illumination plane. Imspector Pro was used for acquiring z-stacks. Image processing and 3D video rendering were performed on arivis Vision4D x64 software (arivis AG, Germany).

Electron microscopy 40 μm thick vibratome sections were blocked and permeabilized with 20% goat serum, 0.1% saponin in PBS. RFP primary antibody (Table 1) was incubated at 4°C for 48h in 20% goat serum, 0.05% saponin in PBS, washed with 0.05% saponin in PBS overnight and secondary antibodies (Table 1) coupled to gold particles (5 nm) incubated, washed and post-fixed for 2h at RT in 2% glutaraldehyde in PBS. Silver enhancement was performed with the R-Gent SE-EM kit (Aurion) and samples post-fixed and contrasted for 1 h on ice with osmium tetroxide 1% and then uranyl acetate 1%. After washing and dehydration with ethanol section were infiltrated and mounted in Epon 812 for 24 h at 60°C and ultrathin microtome sections (UC6 ultramicrotome, Leica Microsystems) collected on formvar-coated grids, stained with uranyl acetate 2% and acquired.

Image processing and analysis Maximum intensity projections of focal planes and mosaic composition were performed using the microscope's optical sectioning system and software. Brain areas, cell numbers and morphological criteria were quantified on digital images using Axiovision (Carl Zeiss),

Photoshop CS5 (Adobe) or Fiji 1.45b (ImageJ). Reconstruction of 4D+ cells in Fig. 3C was done using Amira 6 (Mercury Computer Systems, Inc.). Briefly, the granule cell-specific signal was obtained by thresholding the RFP channel, setting all voxels inside a cell to 1 and all voxels outside to 0. The same was done for all channels and multiplied with the thresholded RFP, resulting in an excised signal representing only RFP+ cells.

Electrophysiology Isoflurane (Forane, Abbvie) anesthetized mice were decapitated and brains transferred to chilled standard artificial cerebro-spinal fluid (ACSF) containing (in mM): 125 NaCl, 2.5 KCl, 25 $NaHCO_3$, 2 $CaCl_2$, 1 $MgCl_2$, 1.25 NaH_2PO_4 and 25 glucose kept in 5% CO_2 and 95% O_2 saturating conditions and pH 7.4. Coronal 300 μm-thick, vibratome OB slices were transferred to a protective ACSF (34°C, 10-15 min) solution containing (in mM): 92 N-methyl-D-glucamine (NMDG), 2.5 KCl, 1.25 NaH_2PO_4, 30 $NaHCO_3$, 20 HEPES, 25 glucose, 2 thiourea, 5 Na-ascorbate, 3 Na-pyruvate, 0.5 $CaCl_2.4H_2O$ and 10 $MgSO_4.7H_2O$, pH 7.4 and subsequently incubated 1 h at room temperature in standard ACSF. Slices were transferred to a recording chamber, superfused with standard ACSF, mounted on a Zeiss microscope (Axio Imager 2, Germany) and cells were visualized in the granule cell layer by epifluorescence. Patch-clamp whole-cell recordings were performed in a total of 12 4D– (GFP+/RFP–) and 10 4D+ (GFP+/RFP+) superficial granule neurons using capillaries (5-10 MΩ) pulled from borosilicate glass (BF150-86-10, Sutter Instruments) in a horizontal puller (P-1000 Micropipette puller, Sutter Instruments) and filled with (in mM): 125 K-gluconate, 5 NaCl, 2 Na_2-ATP, 2 $MgCl_2$, 10 EGTA, 10 HEPES, 10 biocytin at pH 7.4 and containing 0.2 Alexa 488/594 hydrazide (Invitrogen) to allow subsequent

morphological analysis. Current clamp recordings were obtained using a Axopatch 200B amplifier (Molecular Devices), digitized (Digidata 1440A, Molecular Devices), acquired and analyzed with pClamp 10 software (Molecular Devices). Criteria of inclusion were: i) visual confirmation of RFP or GFP fluorescence in the pipette tip, ii) attachment of the labeled soma to the pipette during suction, iii) 4-18 GΩ seal resistances, iv) initial series resistance <40±20% MΩ throughout recordings. Whole-cell capacitance and series resistances were not compensated. In current-clamp recordings, the membrane potential was kept at different values by a holding current. Passive and active membrane properties were recorded by applying a series of hyperpolarizing and depolarizing current steps (10 pA steps, 500 ms). The resting membrane potential (V_{rest}) was estimated from the current-voltage relationship (at I=0). Input resistance (R_{in}) was calculated from the peak of the voltage response to a 50 pA hyperpolarizing 500 ms current step according to Ohm's law (V_{hold}=–60 mV). The membrane capacitance (C_m) was obtained with the membrane time constant equation $\tau m=R_{in}C_m$, where τm was derived from single exponential fitted to voltage response to –50 pA, 500 ms. Rheobase was defined as the amplitude of the first depolarizing step at which firing was observed and was assessed by successive depolarizing current steps (10 pA; 500 ms, V_{hold}=–60 mV). Action potentials were analyzed on the first spike observed at rheobase. Voltage threshold (V_{th}) for spike initiation was defined as the voltage at the inflection point of the initial slope of the action potential. Spike amplitude was measured from threshold to positive peak and after-hyperpolarization (AHP) amplitude, from threshold to negative peak during repolarization. Spike width was measured at the half amplitude. Number of spikes was counted during a 30

pA, 500 ms steps. Frequency and amplitude of the spontaneous excitatory events were recording during 20 s in mode Gap-free and the analysis was done using Clampfit template search. The lag to spiking was measured as the time from the beginning of the depolarizing current pulse and the initiation of the first action potential (V_{hold}=–80 mV). No correction was made for the junction potential between the pipette and the ACSF.

Olfactometry data acquisition and analysis Behavioral tests were conducted with group-housed male mice and in two independent cohorts (n=21 and 17 or 15 and 10, 4D– and 4D+, respectively), with the experimenter blind of genotype. A custom-made automated olfactometer and data acquisition were controlled through custom-programmed software (Igor Pro 6, Wavemetrics Inc.) on mice identified via RFID chips. Animals were trained using odors dissolved in mineral oil at a final concentration of 1%, specifically: cineol (Cin) vs. eugenol (Eu) and subsequently challenged to distinguish pure amylacteate (AA) vs. ethylbutyrate (EB) or (-)-octanol vs (+)-octanol followed by more difficult 60-40% mixtures. In the second cohort Cin vs. Eu testing was omitted while an additional test of a 1:10 diluted binary mixture of the two octanols was included after the binary mixture phase. Odors were pseudo-randomly presented and odor bias avoided by counterbalancing two groups of mice. Briefly, mice voluntarily initiated a trial by introducing their head into the sampling port and breaking an infrared beam. This initiated the opening of the valves connected to the odor reservoirs releasing the odor for 2 s. Trained mice kept their heads inside the port when facing the rewarded odor during these 2 s and started licking afterwards to receive water or, alternatively, retracted their heads from the sampling port

when unrewarded stimuli were presented. All trials were performance-filtered using a rolling average (window size of 100 trials) to calculate the performance for each trial based on the performance in the trial-window directly preceding it. Mice differed in the total number of trials they freely completed with some performing more than 1,000 trials. To account for this, only the first 1,000 trials were compared while excluding mice that completed fewer trials. After reaching the established criterion (≥95% or ≥70% performance) correct trials with a performance at or above criterion were subsequently analyzed by bootstrapping as previously established (Abraham et al., 2010). Specifically, from the pool of available performance-filtered trials, groups of 500 rewarded and unrewarded trials, were randomly sampled with replacement and combined to generate an average sampling rate as a function of time for each group. The two time-dependent curves were subjected to point-wise comparison (using a non-parametric t-test) resulting in a significance value as a function of time. The first time point at which this significance-curve reached p<0,001 was defined as discrimination time (DT) (Fig. EV3B). This resampling and subsequent comparison was repeated 1,000 times with the final DT for each animal representing the average DT of all these resamples.
