# Supplementary material for: An increase in neural stem cells and olfactory bulb adult neurogenesis improves discrimination of highly similar odorants
Source: EMBO J. 2019 Jan 14;38(6):e98791. doi: 10.15252/embj.201798791 (PMC6418468; doi:10.15252/embj.201798791)
Supplement: Supplementary file 2 — Expanded View Figures PDF [file EMBJ-38-e98791-s002.pdf]

Expanded View Figures

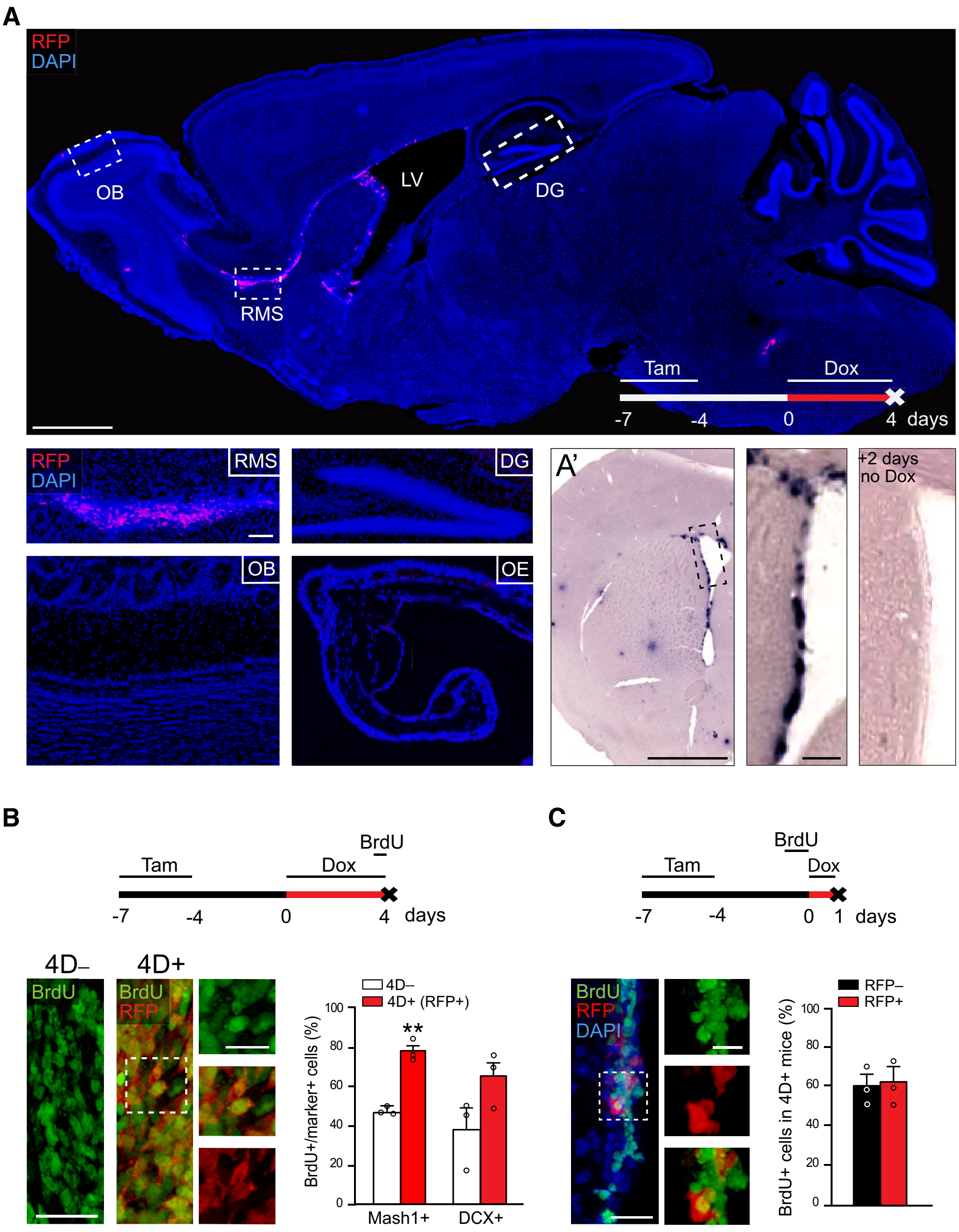

Figure EV1. Characterization of the transgenic model and effect of 4D on the RMS.

A Fluorescence image of a sagittal section of a $4D^+$ brain after a 4-day treatment with doxycycline showing RFP signal confined to the SVZ and RMS (nuclei counterstained with DAPI; blue). Insets show representative images of specific brain regions (as indicated) and the olfactory epithelium.

A' Phase contrast picture of the SVZ upon *in situ* hybridization against mRNA for RFP in a $4D^+$ brain treated as in (A) and sacrificed immediately after (left) or 2 days after (right) doxycycline administration.

B, C Experimental design (top), fluorescence pictures (left with magnified insets), and quantifications (right) of BrdU incorporation in the RMS (B) or SVZ (C). (B) shows the proportion of BrdU in C ($Mash1^+$) and A (DCX^+) cells in $4D^-$ (white) and $4D^+$ (red; among RFP^+) mice. (C) shows the proportion of RFP^- (black) and RFP^+ (red) among $BrdU^+$ cells of $4D^+$ mice. (A) OB, olfactory bulb; RMS, rostral migratory stream; LV, lateral ventricle; DG, dentate gyrus; OE, olfactory epithelium. (A–C) Tam, tamoxifen; Dox, doxycycline. (B, C) Mean ± SEM; $^{**}P < 0.01$; unpaired Student's *t*-test; $N = 3$ mice and $n > 1{,}100$ cells. Scale bars = 500 (A top, A'), 100 (insets A and A'), 50 (B and C), and 20 (insets B and C) µm.

© 2018 The Authors

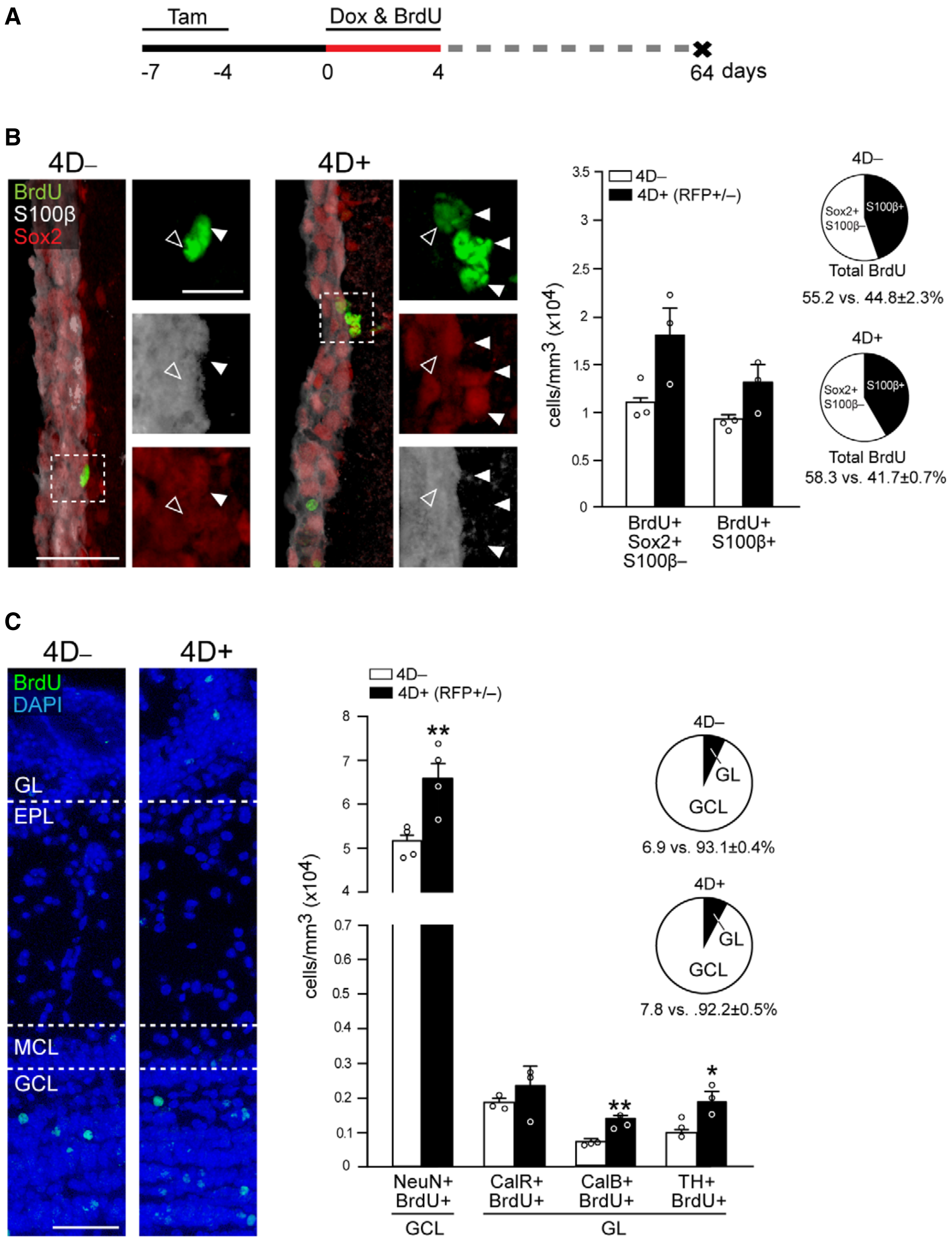

Figure EV2. Long-term effect of 4D overexpression on NSC and OB neurogenesis.

A Experimental paradigm to investigate long-term effects of 4D expression. Tam, tamoxifen; Dox, doxycycline.

B, C From left to right: fluorescence pictures of the SVZ (B) or OB (C) and number per mm^3 (bar graphs) or proportions (pie graphs), of cells in $4D^-$ (left pictures and white bars) or $4D^+$ (right pictures and black bars) mice and scored positive for markers as indicated. Insets in (B) are magnified (right) with arrowheads pointing label-retaining NSC (white) or astrocytes (empty). (C) GL, glomerular; EPL, external plexiform; MCL, mitral cell; and GCL, granule cell layers. (B, C) Mean ± SEM; $*P < 0.05$, $**P < 0.01$; unpaired Student's *t*-test or Fisher's exact test (pie graphs); $N = 4$ mice, $n > 210$ cells. Scale bars = 50 (B and C) and 20 (caption in B) μm.

© 2018 The Authors

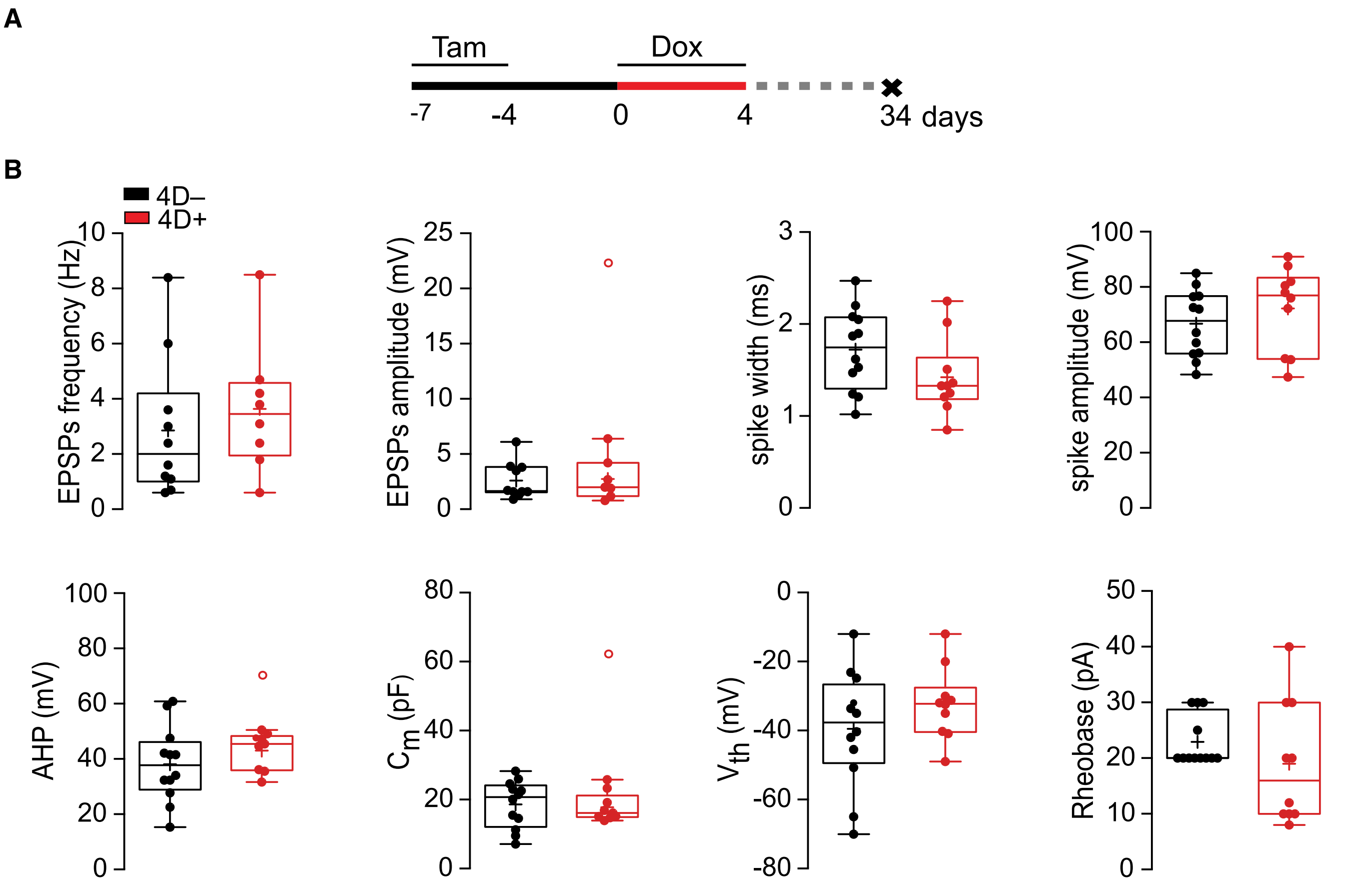

Figure EV3. Electrophysiological parameters.

A Experimental design to assess the integration of 4D-derived neurons.

B Box and whiskers plots extending the electrophysiological analyses of GFP^+/RFP^- (black) and GFP^+/RFP^+ (red) patched superficial granule neurons shown in Fig 3E–H. From left to right: frequency and amplitude of excitatory post-synaptic potential (EPSP), spike width and amplitude (top) and after hyperpolarization (AHP), membrane capacitance (Cm), voltage threshold (Vth), and rheobase (bottom). Significance was calculated by unpaired Student's *t*-test and outliers identified by Tukey's test; $N > 5$ mice, $n > 10–12$ neurons. Scale bars = 10 µm.

 © 2018 The Authors

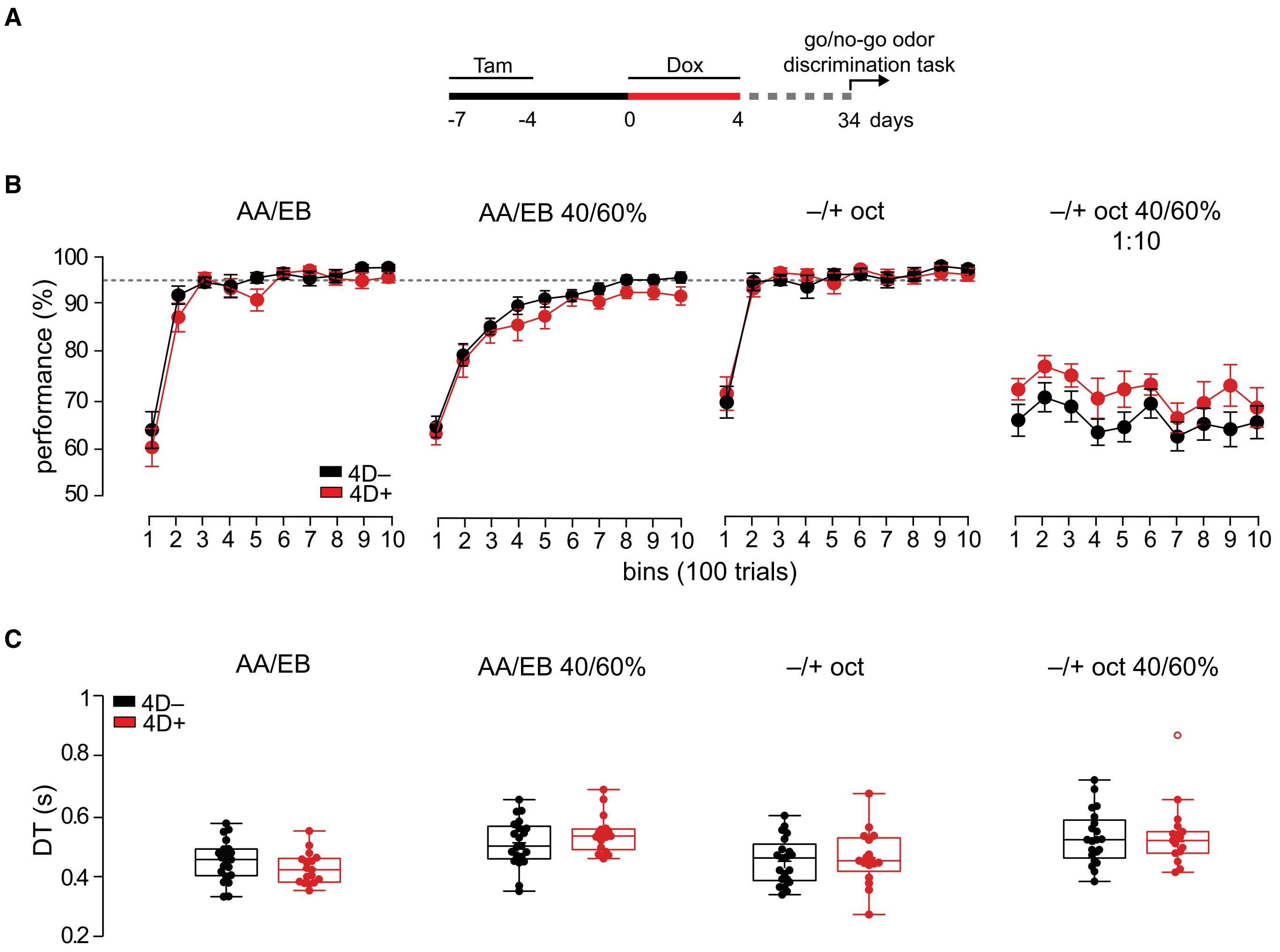

Figure EV4. Performance and DTs at different discrimination tasks.

A Experimental design used to test effects on olfaction. Tam, tamoxifen; Dox, doxycycline.

B Line graphs indicating the proportion of correct responses (performance) for bins of 100 trials during testing with (from left to right): pure and binary mixture of AA vs. EB and pure and 1:10 diluted binary mixture of (−) vs. (+)-octanols. Discontinuous line indicates the similar 95% plateau performance of $4D^{-}$ (black) and $4D^{+}$ (red) mice.

C Box and whiskers plots representing the DTs calculated at 95% performance. Note that DTs are slower using the automated olfactometer compared with the previously used olfactometers owing to differences in odor port design (Abraham *et al*, 2010). (C) AA, amyl acetate; EB, ethyl butyrate; oct, octanol.

Data information: Mean ± SEM with significance tested by repeated measures two-way ANOVA (B) or unpaired Student's *t*-test with outliers identified by Tukey's test (C). Cohorts of N = 21/17 or 15/10 for 1:10 dilution (B; right) $4D^{-}/4D^{+}$ mice were used.

© 2018 The Authors
