# Supplementary material for: An increase in neural stem cells and olfactory bulb adult neurogenesis improves discrimination of highly similar odorants
Source: EMBO J. 2019 Jan 14;38(6):e98791. doi: 10.15252/embj.201798791 (PMC6418468; doi:10.15252/embj.201798791)
Supplement: Supplementary file 3 — Movie EV1 [file EMBJ-38-e98791-s003.zip › MovieEV1/Movie_EV1_legend.rtf]

Movie EV1. Widespread distribution of 4D-derived neurons 3D-reconstruction of the OB of a 4D+ mouse 30 days after doxycycline administration as described in Fig. 3A and subjected to whole-mount RFP immunohistochemistry using a modified version of iDISCO (see SI methods).
